# Antibody Response to SARS-CoV-2 Vaccines in Transplant Recipients and Hemodialysis Patients: Data from the Dominican Republic

**DOI:** 10.3390/vaccines12121312

**Published:** 2024-11-23

**Authors:** Lisette Alcantara Sanchez, Eloy Alvarez Guerra, Dongmei Li, Samantha M. King, Shannon P. Hilchey, Qian Zhou, Stephen Dewhurst, Kevin Fiscella, Martin S. Zand

**Affiliations:** 1 Clinical and Translational Science Institute, University of Rochester, Rochester, NY 14642, USA; lisette_alcantara@urmc.rochester.edu (L.A.S.);; 2 Instituto Nacional de Coordinación de Trasplante, Instituto de Medicina Tropical y Salud Global, Universidad Iberoamericana, Santo Domingo 01219, Dominican Republic; e.alvarez1@prof.unibe.edu.do; 3 Department of Medicine, Division of Nephrology, University of Rochester, Rochester, NY 14642, USAqian_zhou@urmc.rochester.edu (Q.Z.); 4 Department Microbiology and Immunology, University of Rochester, Rochester, NY 14642, USA; 5 Department of Family Medicine, University of Rochester, Rochester, NY 14642, USA; kevin_fiscella@urmc.rochester.edu; 6 Department of Public Health Sciences, University of Rochester, Rochester, NY 14642, USA

**Keywords:** COVID-19, SARS-CoV-2 vaccine, transplant recipients, hemodialysis, immunocompromised, antibody response, anti-S, anti-N

## Abstract

**Background**: The global COVID-19 pandemic has resulted in approximately 7 million deaths and a historic vaccination effort, with over 13.6 billion doses administered. Despite this, understanding of immune responses in vulnerable populations, such as transplant recipients (TR) and hemodialysis patients (HD), remains limited, especially outside the US and Europe. **Methods**: To address this gap, we analyzed blood samples and deidentified data from the Instituto Nacional de Coordinación de Trasplante (INCORT) in The Dominican Republic, measuring antibody levels to SARS-CoV-2 post-infection and vaccination with BNT162b2 (Pfizer-BioNTech) and Sinovac-CoronaVac (Sinovac) in TR, HD, and healthy controls (CO). Using a fluorescent multiplex assay (mPlex-CoV) and mixed-effects modeling, we assessed variations in anti-S, anti-RBD, and anti-N IgG antibodies. **Results**: The results indicate that the CO group experienced an early peak in anti-S and anti-RBD antibodies, followed by stabilization. In contrast, the TR and HD groups showed a slower, gradual increase in antibodies. Despite fluctuations in the HD group, both the TR and HD groups maintained high anti-S and anti-RBD IgG levels, indicating a back-boosting effect from vaccination. However, elevated anti-N IgG levels in the TR and HD groups suggest potential reinfections. Additionally, prior SARS-CoV-2 infection led to higher anti-S IgG levels, with BNT162b2 associated with higher anti-S IgG and CoronaVac associated with higher anti-N IgG levels. **Conclusion**: These findings highlight the variability in antibody responses and the need for targeted public health strategies to diverse immunological profiles.

## 1. Introduction

The coronavirus disease 2019 (COVID-19) global pandemic has resulted in approximately 7 million deaths [1]. The key to managing this public health crisis has been vaccination against severe acute respiratory syndrome coronavirus 2 (SARS-CoV-2). Several vaccines with a variety of platforms have been approved for use worldwide, including inactivated viral vector, nucleoside-modified mRNA, and recombinant-expressed protein vaccines [2]. As of August 2024, over 13.6 billion vaccine doses have been administered across the world [1]. However, there are still sparse data regarding the dynamics of the antibody-mediated immune response to COVID-19 vaccines, especially the intensity and duration of antibodies. There are modest data regarding protective SARS-CoV-2 immunoglobulin G (IgG) levels in immunocompromised populations originally excluded from clinical trials, such as transplant recipients (TR) and hemodialysis patients (HD) [3,4]. This was particularly true in many countries outside of the United States and Europe, where vaccine supplies varied in terms of availability, preparations, and delivery logistics [5].

Antibodies that bind to the SARS-CoV-2 spike protein receptor binding domain (RBD), arising from either infection or vaccination, are considered a surrogate of immune protection [6,7,8]. The vaccination results in SARS-CoV-2 anti-spike (anti-S) protein receptor-binding domain (S-RBD) antibodies demonstrated seroconversion rates of 81% in hemodialysis (HD) patients with prior infection and 77% in those without [9]. However, studies suggest a substantial decrease in anti-S SARS-CoV-2 immunoglobulin G (IgG) titers three months after infection in patients with chronic kidney disease [10]. Data for non-SARS-CoV-2 vaccines (e.g., hepatitis B, influenza, Clostridium tetani, Corynebacterium diphtheriae)suggest that vaccine-induced IgG in patients with end-stage kidney disease can vary substantially with respect to neutralizing antibody titers and the duration of pathogen specific immunity [3,10,11,12,13].

Chronic kidney disease (CKD) significantly increases the risk and severity of COVID-19, especially among HD patients, who have increased morbidity and mortality from infection [10,14]. In England, patients on in-center HD experienced an 11.3% infection rate and a 23% fatality rate; far higher than that of age-matched, non-hemodialysis subjects [15]. Similarly, TR patients also face unique challenges in generating an effective antibody response to infections and vaccines [16]. One study from 2021 of SARS-CoV-2 mRNA vaccines from 2021 in TR reported that only 17% of subjects had increased SARS-CoV-2 anti-S IgG levels after a single vaccine dose [12,16], while 58% sustained low seroconversion rates even after a second [16,17] or third vaccine dose [18]. These low seroconversion rates may be due to the prolonged use of immunosuppressive medications, along with an increased prevalence of comorbidities such as diabetes and cardiovascular disease [16,18,19,20]. Low levels of anti-spike IgG following a third vaccine dose in kidney transplant recipients were associated with symptomatic breakthrough SARS-CoV-2 infections and hospitalizations. Specifically, low anti-RBD IgG levels were significantly correlated with hospitalization during the Omicron surge [21]. While more vaccine booster doses appear to improve outcomes such as disease severity, hospitalization, and mortality rates, HD [22] and TR [21,23,24,25] continue to experience high rates of breakthrough infections despite multiple doses of COVID-19 vaccines. Taken together, these findings suggest a persistent high risk of COVID-19 in CKD and TR despite vaccination.

The Dominican Republic (DR) has a substantial burden of chronic kidney disease, with more than 7080 individuals reported in 2023 [26]. Of these, approximately 5275 were undergoing dialysis, and 4083 were receiving hemodialysis [26]. Additionally, 500 individuals were awaiting a kidney transplant, while about 299 received a kidney transplant between 2019 and 2023 [27]. It is crucial to generate data on COVID-19 vaccine responses in HD and TR in the DR to understand how demographic, environmental, and healthcare factors influence vaccine efficacy, ultimately enhancing healthcare outcomes both locally and globally. The DR’s national COVID-19 vaccination program began in February 2021, prioritizing individuals at high risk of infection and mortality, including HD and TR [28]. Three vaccine options—BNT162b2 (Pfizer-BioNTech), AZD1222 (AstraZeneca—Oxford), and CoronaVac (Sinovac)—were made available [29]. By March 2024, approximately 15 million doses had been administered to an estimated population of 11 million individuals, with 68% of the population receiving at least one dose and 56% being fully vaccinated with two or more doses [1]. However, there are limited real-world or clinical trial data on vaccine responses to HD and TR patients in the DR. This is critical because real-world variation in vaccine responses may occur due to variations in local and population health that are particular to individual countries (e.g., vaccine type available, access, ease of reaching populations).

To generate data that might facilitate a better understanding of how to protect vulnerable populations in real-world circumstances, we measured the IgG anti-S and anti-N immune response to SARS-CoV-2 infection and vaccination in TR and HD patients in the Dominican Republic. We performed a secondary analysis of capillary blood samples collected from Dominican HD and TR patients via volumetric micro sampling (VAMS) obtained from a previously approved study conducted by Instituto Nacional de Coordinación de Trasplante (INCORT). IgG levels against SARS-CoV-2 spike protein and S-RBD, induced by both infection and vaccination among TR, HD, and healthy control subjects (CO), were measured using multiplex analysis, allowing for simultaneous detection of antibodies directed at the S- and N-proteins of SARS-CoV-2 strains [6,30,31].

## 2. Materials and Methods

### 2.1. Human Subjects Ethics Statement

This is a secondary analysis of existing capillary blood samples and deidentified data previously obtained by the INCORT in the Dominican Republic. The protocol was approved by the National Health Bioethics Committee of the Dominican Republic (CONABIOS, the Spanish acronym) (INCORT.PI2020.004.01). The secondary analysis was approved by the Institutional Review Boards (IRB) at the University of Rochester Medical Center (IRB STUDY00006807). All research data were coded in compliance with the Department of Health and Human Services Regulations for the Protection of Human Subjects (45 CFR 46.101(b) (4)).

### 2.2. Subject Data Analysis

The INCORT parent study enrolled 70 subjects sourced from four hospitals that are part of the national registry of transplant recipients, as well as from the dialysis units of three national referral hospitals. Additionally, a control group was recruited at three COVID-19 vaccination clinics across Santo Domingo, DR. Medical personnel and study staff disseminated information and recruited patients into the parent study through phone calls, during in-person hemodialysis sessions, and via follow-up visits for transplant recipients.

**Figure 1:**
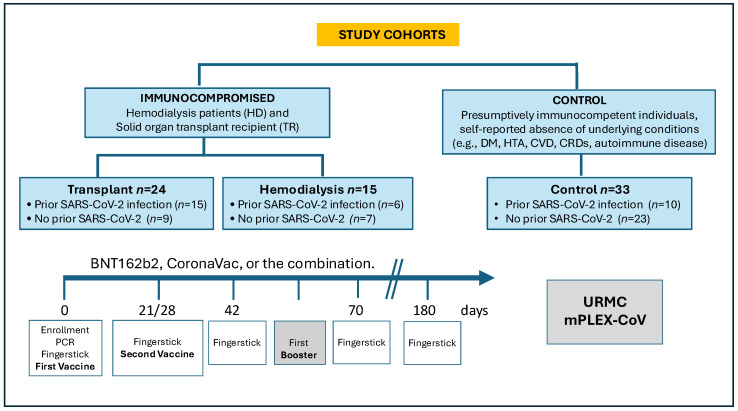
Data flow diagram showing study cohorts and design. We studied 271 capillary blood samples from 70 subjects at 5 time points after vaccination. Three cohorts were considered: control (*n* = 33), hemodialysis (*n* = 13), and transplant (*n* = 24), stratified by prior history of COVID-19 infection. Day 0 corresponds to the samples that were collected immediately prior to the first vaccine, and day 21/28 indicates the samples collected prior to the second dose, depending on the vaccine schedule. Additional samples had been collected on days 42, 70 (2 and 4 weeks after the second vaccine dose), and before any booster applications, if administered, as well as 6 months after the last received dose.

The eligibility criteria included adults aged 18 years or older who were undergoing hemodialysis or were solid organ transplant recipients willing to receive a COVID-19 vaccine. The control group comprised individuals who reported the absence of comorbidities, including prevalent conditions such as hypertension, diabetes mellitus, cardiovascular disease, chronic respiratory disease, immunodeficiency, and degenerative and autoimmune diseases (Figure 1). To establish a well-defined reference group for comparison with the two cohorts with histories of immunosuppression, dialysis, and organ transplantation, this study included a control group free from common medical conditions known to increase the risk of COVID-19 infection and severe illness [32].

This study excluded individuals who tested positive for COVID-19 via polymerase chain reaction (PCR) at enrollment or on day 0, as well as those who had received any prior dose of a COVID-19 vaccine. This focus on unvaccinated groups aimed to establish a homogeneous study population, enabling a comprehensive analysis of key variables within the unique context of dialysis, organ transplantation, and COVID-19 vaccination status. Secondary analysis also included data previously collected by INCORT via in-person interviews by the INCORT research staff based on a self-reported questionnaire including demographics, health status, and history of COVID-19 infection and vaccination. The specific data included age, sex, race, ethnicity, highest level of education achieved, current medication, vaccine dosage, and immunization history (Hepatitis B, influenza). For those with a history of SARS-CoV-2 infection, the data included the severity of COVID-19 symptoms, including hospitalization, intensive care, and mechanical ventilation needs. The clinical variables in the HD and TR cohorts included the duration and type of dialysis access, time since transplantation, history of organ rejection, and details of both induction and current immunosuppression therapy. The clinical variables are summarized in (Tables S1–S3). We utilized this comprehensive collection of variables in generalized linear modeling to enable a nuanced analysis of the complex interactions among organ transplantation, dialysis, COVID-19 infection, vaccination status, and other health factors within the study cohort. The cohorts’ demographics, infection, and vaccination status are summarized in (Table 1 and Table 2).

### 2.3. Sample Collection

The secondary analysis included 271 volumetric absorptive microsampling (VAMS) capillary blood samples previously obtained from 70 subjects prior to SARS-CoV-2 vaccination and obtained after receiving at least one dose of either the BNT162b2 (Pfizer-BionTech) mRNA or CoronaVac (Sinovac) inactivated virus COVID-19 vaccine. This included both homologous and heterologous boosters, with the choice of vaccine determined by adherence to the Dominican Republic national guidelines for COVID-19 vaccination [28]. Heterologous boosters involve using a different vaccine for the booster dose, while homologous boosters use the same vaccine as the primary vaccination. The study population included three cohorts, each consisting of transplant recipients (TR), hemodialysis patients (HD), and healthy controls (CO). Each cohort was additionally stratified by subjects with a reported history of infection with, or testing positive for, SARS-CoV-2 infection, and those without this history. Samples previously banked by the INCORT in the Dominican Republic were sent to the University of Rochester Medical Center analysis of SARS-CoV-2 IgG-mediated immunity based on a mPlex assay [6,30,31].

### 2.4. mPlex-CoV Assay

The samples were batch-processed as previously described [6,30,31]. Antibodies were eluted from the VAMS swabs by soaking them in 200 μL of extraction buffer (0.5% Tween 20, 1% BSA in PBS buffer) at 4 °C under gentle agitation overnight. The eluents were aliquoted into 100 μL tubes and stored at 4 °C for the multiplex assay [33]. We used the mPlex-CoV multiplex assay to measure IgG concentrations directed against SARS-CoV-2 spike (S) and nucleocapsid (N) protein antigens from multiple viral variants [6,30,31]. The targets included recombinant, trimerized SARS-CoV-2 S- and N- proteins, along with recombinant S1, S2, and RBD domain proteins (SinoBio, Beijing, China). The proteins were coupled to magnetic microsphere beads (Luminex, Austin, TX, USA) at 40 pmole/106 beads using the xMAP^®^ Antibody Coupling kit (Luminex, Austin, TX, USA). For the assay, 200 μL of eluent from VAMS devices (diluted at 1:20) underwent further dilution at 1:50 (for the IgG assay), resulting in final sample dilutions of 1:1000 (IgG). A total of 50 μL of diluted sample was added to duplicate wells of black, clear-bottomed 96-well plates (Microplate, GBO, Kremsmünster, Austria), with 50 μL of the mPlex-CoV bead panel added to each well. After washing, the bound IgG was detected using phycoerythrin-conjugated anti-human IgG (Southern Biotech, Birmingham, AL, USA, Cal No:2040-09, 2050-09), and the median fluorescence intensity (MFI) was read using a Luminex MagPix [6,31].

### 2.5. Measurement of Hemoglobin (Hgb) and Adjustment of Antibody Concentration

We adjusted capillary blood IgG concentrations for plasma volume using Hgb measured from the VAMS eluent samples, as previously described [33]. Hematocrit was calculated from Hgb concentrations to adjust the measured SARS-CoV-2 IgG concentration to estimate serum IgG concentrations. Finally, the concentrations of specific SARS-CoV-2 S (S1, S2, RBB) and N IgG antibodies in each sample were estimated using standard IgG curves generated from positive pooled sera samples, as previously described [6,31].

### 2.6. Statistical Analysis

The participant characteristics were summarized using frequency distributions and summary statistics. Differences between study groups were assessed using either the Chi-square or Fisher’s exact test for categorical variables. The non-parametric Kolmogorov–Smirnov test was applied to assess whether the antibody levels were normally distributed across different sample batches. Generalized linear mixed effects modeling (GLMM) was used to assess the longitudinal anti-S, S1, S2, RBD, and anti-N SARS-CoV-2-specific IgG antibody response elicited by COVID-19 vaccination and infection over time; factors associated with changes in antibody concentrations; and differences between cohorts. Pearson’s correlation coefficient was applied to assess the association between the responses of the different IgG antibodies and between cohorts.

We used binary logistic regression to examine the independent associations between predictors and the primary outcome, SARS-CoV-2 IgG antibodies [34]. We assessed multicollinearity by calculating Variance Inflation Factors (VIFs), with all predictors having VIF values below the threshold of 5. The goodness-of-fit of the GLMM models was examined using residual diagnostic plots. We developed distinct GLMMs to analyze the anti-S, anti-RBD, and anti-N IgG responses, ensuring that each model consistently included demographic variables, prior COVID-19 infection history, and vaccine-related factors such as vaccine type and number of doses. We fitted the models using the glmmTMB function in R, incorporating splines to more accurately capture significant changes at key points along the timeline. Statistical analysis was performed using the statistical analysis software R, version 3.5.1 (R Core Team, 2017). The significance level for all the tests was set to *p* < 0.05.

## 3. Results

### 3.1. Participant Characteristics

The subject demographics are shown in Table 1, including 70 subjects distributed across three cohorts: control (*n* = 33, 59%), HD (*n* = 13, 18%), and TR (*n* = 24, 23%). There were no statistically significant differences in age or gender between the cohorts (*p* = 0.150), although there was a slightly higher percentage of male subjects (57%) compared to females across the HD and TR cohorts, but not in the CO cohort. The majority of the subjects identified as Black and Hispanic or Latino (*p* < 0.001). This is consistent with the demographics in the Dominican Republic, where 74% of Dominicans identify as mixed race [35]. The DR distinguishes between race and ethnicity in a way that reflects the country’s unique historical and social context, unlike in the US, where race and ethnicity are often treated as separate categories. However, it is important to note that the questionnaire used in our study did not include mixed race as an option, which may have influenced respondents who identified with this category to choose Black as their predominant race.

With respect to education, the TR cohort had a larger proportion of subjects with higher educational levels (*p* < 0.001). As expected, subjects without underlying conditions were primarily in the CO group, while those with at least one underlying medical condition were more evenly distributed across the cohorts. Additionally, 44% of the subjects reported a history of COVID-19 infection or a positive test before the initial sample and vaccination (Table 1). The imbalance in recruitment between the cohorts resulted from challenges faced during INCORT recruitment. Initially, accelerated vaccination efforts prioritized high-risk groups, including hemodialysis and transplant recipients, making it difficult to find unvaccinated individuals.

### 3.2. Vaccination Characteristics and Prior COVID-19 Status

We compared vaccine and previous infection characteristics across the three cohorts. (Table 2) We found significant differences in the number of vaccine doses (*p* < 0.001), with the TR cohort having the highest percentage of fully vaccinated individuals with one booster (42%). Although variation was present in vaccine type distribution between the groups, these differences did not reach statistical significance (*p* = 0.066). Similarly, differences in booster combinations with respect to the primary vaccine (homologous or heterologous) were also not statistically significant. In all cases, for heterologous vaccination, the initial vaccine administered was CoronaVac, with a subsequent BNT162b2 booster. Conversely, in the homologous group, the trend was use of both a BNT162b2 prime and booster (87.5%), with only one participant opting for CoronaVac (12.5%).

Regarding previous COVID-19 infection, while the TR cohort had a higher percentage of previously positive COVID infection or positive PCR tests (63%) compared to HD (46%) and CO (30%), these results did not reach statistical significance. Similarly, there was no statistically significant difference between cohorts in COVID-19 positivity during this study, though most data were not reported, as only 21.43% of the subjects reported being aware of testing positive prior to the study.

### 3.3. Generalized Linear Modeling

To determine the association between SARS-CoV-2 anti-S antibody concentrations and other variables, we first performed a simple linear regression analysis to aid in variable selection. We found that age (*p* = 0.001), the male sex (*p* = 0.043), years of education (*p* = 0.0003), and the number of medical conditions reported (*p* = 0.064) had significant positive associations with SARS-CoV-2 anti-S IgG concentrations across all sampling time points.

Subsequently, we conducted a generalized linear model analysis, including variables that showed a positive association (*p* < 0.100) with SARS-CoV-2 anti-S IgG in the simple linear regression analysis (see Table 3 for β values, 95% confidence intervals, and *p*-values). COVID-19 infection before the first vaccine was associated with increased anti-S IgG levels (*p* = 0.014). Similarly, receiving CoronaVac as the first vaccine dose (*p* < 0.001) and the number of doses received played a significant role, showing a positive association with elevated anti-S IgG levels (*p* = 0.008). Furthermore, receiving a homologous booster schedule was significantly associated with higher anti-S IgG levels (*p* = 0.014). As noted above, the homologous vaccination group predominantly received BNT162b2 for both the initial vaccination and the booster (Table 2), while the heterologous group received CoronaVac followed by a BNT162b2 booster. Heterologous boosters enhance immunogenicity, broaden protection against variants, improve antibody responses, and boost T-cell immunity, especially in transplant recipients [36,37]. This approach has been shown to provide extended protection beyond antibodies, potentially offering longer-lasting defense against SARS-CoV-2 with fewer adverse effects [37].

Samples collected 14 and 30 days after the second dose, as well as six months after the final dose (time points 3–5), shoedw statistically significant elevations in SARS-CoV-2 anti-S IgG levels of 0.63 Log ng/mL (95% CI: 0.17–1.10; *p* = 0.008), 0.54 Log ng/mL (95% CI: 0.10–0.98) (*p* = 0.018), and 0.90 Log ng/mL (95% CI: 0.43–1.4), respectively. As anticipated, these results suggest that later time points are associated with higher SARS-CoV-2 anti-S IgG levels compared to earlier time points. Lastly, the HD cohort had a greater association with higher SARS-CoV-2 anti-S IgG levels (β= 1.3, 95% CI: −0.13–2.80) *p* = 0.073) across all time points when compared with the other two groups.

### 3.4. Time Varying Anti-SARS-CoV-2 IgG Response

**Figure 2:**
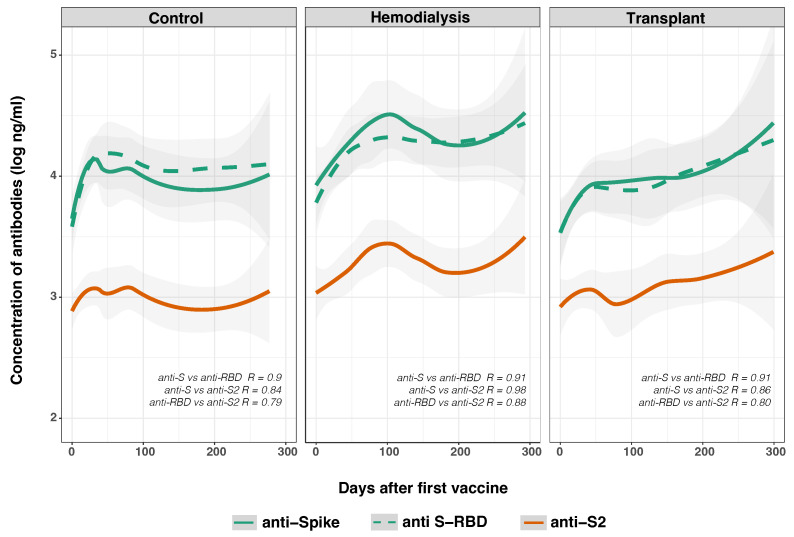
Total full-length anti-spike (green solid line), S-RBD (green dashed line), and S2 (orange solid line) subunit IgG concentrations were measured in control (*n* = 33), hemodialysis (*n* = 13), and transplant recipient (*n* = 24) cohorts. Day 0 corresponds to the day of the first vaccine dose. Data were fitted to a spline curve for each group, with 95% confidence levels noted as gray bands. Significant differences in antibody concentrations between time points, defined as $0.2\log_{10}$ ng/mL, were detected based on the GLMM analysis and determined by *p*-values < 0.05). Statistically significant correlations were found between the different subunits of the spike protein, with group-specific R values.

We evaluated the development of the anti-SARS-CoV-2 spike IgG antibody resulting from either SARS-CoV-2 infection and/or vaccination. IgG concentrations were estimated against the original Wuhan variant SARS-CoV-2 full S protein and subunits (S1, S2, RBD) before and after the administration of either BNT162b2 or CoronaVac vaccines (Figure 2). As hypothesized, there was a strong positive correlation between concentrations of anti-spike SARS-CoV-2 IgG and both anti-S2 IgG and anti-RBD IgG across all time points. While the association between increased anti-S IgG and anti-RBD IgG was more robust (R = 0.92) (Supplementary Figure S1), a strong correlation was found with anti-S2 IgG levels (R = 0.86) (Supplementary Figure S2). Anti-S2 IgGs have been reported to correlate with viral neutralization [38]. Furthermore, the concentrations of SARS-CoV-2 IgG against the S-RBD and S2 subunits also exhibited a statistically significant correlation (R = 0.81) (Supplementary Figure S3).

We observed statistically significant changes in anti-S IgG antibody levels (*p* = 0.028) across all time points after adjusting for the variables included in the GLMM (Table 3). The most pronounced differences occurred on day 130 (*p* < 0.001), regardless of the cohort. Notably, the HD group (4.08 ± 0.587 log ng/mL) and the CO group (3.82 ± 0.717 log ng/mL) exhibited higher antibody concentrations compared to the TR group (3.79 ± 0.859 log ng/mL). Statistically significant differences were also noted in anti-S-RBD IgG antibody concentrations (*p* = 0.041), with higher levels in the HD (4.01 ± 0.566 log ng/mL) and CO groups (3.88 ± 0.690 log ng/mL) compared to the TR group (3.73 ± 0.830 log ng/mL). Additionally, differences were found in anti-S2 IgG antibody concentrations (*p* = 0.006) between the HD group (3.11 ± 0.447 log ng/mL) and CO group (2.86 log ± 0.553 log ng/mL), both greater than the TR group (2.84 ± 0.778 log ng/mL) by a slight margin.

As shown in Figure 2, in the CO group, anti-S IgG levels increases to ∼4 log ng/mL by day 50, remaining steady through day 300, with anti-RBD IgG following a similar pattern at lower levels. In contrast, anti-S IgG in the HD group shows a steady increase to ∼5 log ng/mL by day 300, with anti-RBD also rising above 5 log ng/mL, while anti-S2 remained stable at ∼3 log ng/mL. The TR group started with lower anti-S and anti-RBD IgG levels, but both increased significantly over time to ∼5 log ng/mL by day 300, and anti-S2 rose to just below ∼3 log ng/mL. These results suggest that while the antibody response in the CO group stabilized, the HD and TR groups experienced a delayed but continuous increase in SARS-CoV-2 S IgG antibody concentration over time, indicating differences in immune response dynamics between the groups.

### 3.5. SARS-CoV-2 Anti-Spike and Anti-Nucleocapsid IgG Responses

**Figure 3:**
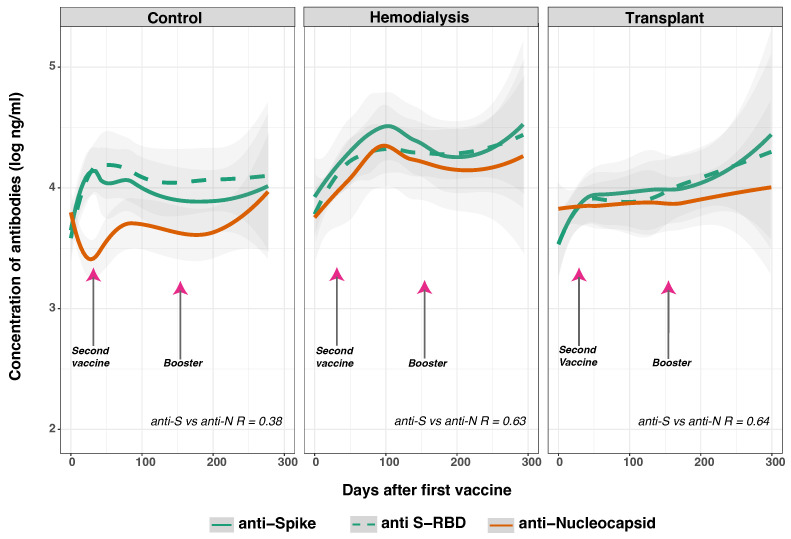
SARS-CoV-2 IgG anti-S and anti-N IgG distribution estimated by days after first vaccine dose. Total anti-S (green solid line), anti S-RBD (green dashed line), and anti-N (orange solid line) IgG concentrations for control (*n* = 104), hemodialysis (*n* = 47), and transplant (*n* = 120) cohorts were measured using a multiplex assay. Day 0 corresponds to the day of the first vaccine dose. The data were fitted to a spline curve for each group, with 95% confidence levels noted by the gray bands. Arrows indicate the mean time for the second (36.6 ± 34.1 days) and booster vaccinations (154.3 ± 41.1 days). The correlation between anti-S and anti-N over time is R = 0.54, with group-specific R values displayed in the figure. A statistically significant difference in anti-S levels was observed between the HD and CO groups (*p* < 0.001), with the most notable difference occurring on day 130 after the first vaccine dose (*p* = 0.014). In CO, the anti-N levels increased after 150 days, while, unexpectedly, anti-S IgG declined compared to the levels observed on day 50 (*p* < 0.001). This pattern suggests a potential increase in infections during the first 130 days after the first vaccine. Conversely, the TR group exhibited a statistically significant (*p* < 0.05) delayed yet continuous increase in anti-S levels, beginning around day 65. This trend suggests a prolonged immune response and potentially greater variability in this group. Follow-up for the control group was limited to 300 days, with some subject dropout (see Table 3).

We next assessed immunologic evidence of SARS-CoV-2 infection and vaccination during the study period among the three cohorts by measuring the levels of anti-S, anti-RBD, and anti-N IgG against the SARS-CoV-2 Wuhan variant (Figure 3). Others have reported that for the general population, the anti-S IgGs peak between 22 and 28 days after infection or vaccination [39], maintaining levels that correlate with viral neutralization up to 180 days from the peak response and showing a modest decrease over time [40]. We hypothesized that we would observe blunted responses in the HD and TR populations, anticipating earlier and accelerated declines in peak levels of anti-S and anti-RBD IgGs compared to the CO group.

After receiving two vaccine doses (36.6 ± 34.1 days), a total of 244 samples from the CO (*n* = 93), HD (*n* = 37), and TR (*n* = 114) groups were analyzed. Our analysis showed statistically significant differences in both anti-S and anti-RBD IgG levels between CO and HD (*p* < 0.010), as well as between CO and TR (*p* < 0.050) from day 0 to day 130 after the first dose. The CO group exhibited an increase in both anti-S and anti-RBD IgG levels, beginning on day 25 and peaking at day 50 (*p* < 0.030). Similarly, the HD group showed an increase in both anti-S IgG levels (1.416 log ng/mL) and anti-N IgG levels (1.01712 log ng/mL), with a moderate correlation (R = 0.63) (Supplemental Figure S4). Comparable trends were observed in the TR group (R = 0.64), but the anti-S IgG antibody levels in the TR group were significantly lower compared to the CO group (*p* < 0.050) and the HD group (*p* < 0.030). This observation in TR aligns with existing literature highlighting a diminished vaccine immune response in these immunosuppressed individuals [41].

The extended follow-up period in the HD group, and differences in the number of doses between groups, may contribute to the sustained increase in anti-S IgG. A significant proportion (79%, *p* < 0.001) of the HD group received two or more doses, which was slightly lower than the control group’s rate (93%). Additionally, 23% of the HD group received booster doses, compared to 9% in the CO group. Compared to the CO group, the HD cohort had weaker and faster-declining immune responses to the standard two-dose mRNA COVID-19 vaccines [10,22]. While strategies like adjusting vaccine doses and timing are being studied, emerging variants have led to recommendations of multiple boosters, as well as bivalent vaccines targeting both the original strain and emerging variants of concern (e.g., omicron) [42]. It is essential to emphasize that 21% of the HD group, in contrast to 7% of the CO group, are excluded from Figure 3, as they received only one vaccine dose (see Table 2).

The antibody trends for anti-S and anti-RBD IgG varied across groups over time. In CO, anti-S antibodies peaked on day 35 (*p* < 0.001), and anti-RBD antibodies peaked on day 50 (*p* = 0.002), followed by stabilization. In contrast, both anti-S and anti-RBD IgG concentrations rose slowly in the TR group, peaking after ∼250 days (anti-S IgG: *p* = 0.006; anti-RBD IgG: *p* = 0.025). The HD group showed an initial increase in anti-RBD IgG levels, peaking around day 65 (*p* = 0.059), followed by consistently high levels throughout the study. The pattern of anti-RBD IgG observed in the TR and HD groups suggests that the increase is likely due to SARS-CoV-2 vaccination rather than reinfection. This is consistent with reports that anti-RBD IgG levels remain stable with reinfection but rise with vaccination [43]. This phenomenon of back boosting occurs when a subsequent vaccination or infection with a different strain of the virus enhances the immune response against previously encountered strains, and is facilitated by reactivation of memory B and T cells initially generated in response to earlier exposure to antigenically similar strains [44].

A comparative analysis of anti-N IgG concentrations showed distinct patterns in the HD and TR groups compared to the CO group. Although both TR and HD had consistently higher anti-N IgG levels than the CO group (*p* = 0.003), a later increase in anti-N IgG levels in the CO group points to potential new infections. In TR, anti-N IgG levels remained relatively constant. In the HD group, anti-N IgG levels peaked at ∼100 (*p* = 0.056), followed by a decline starting on day 130 (*p* = 0.017). In contrast, the CO group had consistently low anti-N IgG levels until day 200, after which a significant increase was observed (*p* = 0.012). This rise in anti-N IgG in the CO group, coinciding with a decline in anti-S IgG, suggests potential new infections. This observation is consistent with existing literature showing that anti-N IgG antibodies usually peak around 100 days after symptom onset and can persist for 200–550 days after infection [43]. Those without reinfection show early waning of anti-N IgG levels, while individuals with reinfection experience an increase in anti-N IgG levels (*p* < 0.0001). Vaccination does not significantly affect anti-N IgG levels over time (*p* = 0.649) [43]. In the next section, we explore prior infection and different vaccine types as potential factors influencing antibody dynamics post-SARS-CoV-2 vaccination and potential infections.

### 3.6. SARS-CoV-2 Anti-Spike and Anti-Nucleocapsid IgG Response in Previously Infected and Non-Infected Subjects

**Figure 4:**
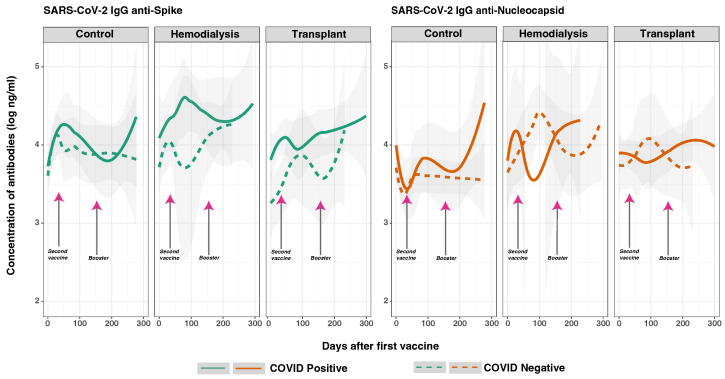
Distribution of SARS-CoV-2 IgG antibodies against spike and nucleocapsid proteins by cohort and COVID-19 status before vaccination. Concentrations of total anti-S (green lines on the left) and anti-N (orange lines on the right) IgG measured in the three studied groups after first vaccine dose. Solid lines represent subjects with a reported prior natural infection (*n* = 31), while dashed lines represent those without a history of COVID-19 infection (*n* = 39). Day 0 corresponds to the day of their first vaccine dose. Aggregate data points were fitted to a spline curve for each group, with 95% confidence levels noted by the gray bands. Arrows indicate mean times for second (36.6 ± 34.1 days) and booster vaccinations (154.3 ± 41.1 days).

To study the effects of prior SARS-CoV-2 infection on post-vaccination anti-S and anti-N IgG levels, we measured anti-S and anti-N IgG concentrations after the first vaccine dose (Figure 4). We hypothesized that, compared to subjects without prior SARS-CoV-2 infection, subjects who had a history of prior SARS-CoV-2 infection would have higher and sustained anti-S IgG levels post-vaccination, regardless of their immunocompromised status. The GLMM analysis indicated that prior COVID-19 infection before the first study sample continued to alter subsequent anti-S IgG levels over time (Table 3). As expected, the subjects who had acquired natural immunity from a previous COVID-19 infection showed significantly higher levels of anti-S IgG post-vaccination compared to those without a history of infection. This trend was observed across all groups, with CO and HD displaying more pronounced differences compared to TR. In contrast, the anti-N IgG levels in previously infected patients overlapped with those of patients without prior infection across these groups.

When comparing responses based on prior infection status, significant differences were observed in the overall response based on anti-S IgG (*p* < 0.001) and anti-N IgG levels (*p* < 0.050). Notably, the overall response based on anti-S2 IgG levels was also significantly higher in patients with prior infection (*p* < 0.001). Among subjects with a history of COVID-19 infection, anti-S IgG levels were significantly higher in the HD group compared to the CO group (*p* < 0.010), though no significant differences were found when compared to the TR group (*p* > 0.050). Interestingly, anti-N IgG levels were significantly lower in the TR group compared to the CO group (*p* < 0.050), but no significant differences were observed between the CO and HD groups.

As we hypothesized, individuals with a history of prior COVID-19 infection had significantly higher initial anti-N IgG levels compared to those without a history (*p* = 0.014). In the CO and HD groups, the initial levels were ∼4.5 log ng/mL, while in the TR group, the levels were ∼3.5 log ng/mL. By day 100, these levels increased to 5 log ng/mL for the CO and HD groups and then stabilized to ∼4.5 log ng/mL through the end of the follow-up period, reflecting a sustained immune response. In contrast, the TR subjects had slightly lower levels, with minor fluctuations, stabilizing at ∼3.5 log ng/mL. The literature on anti-N IgG antibody levels following the CoronaVac vaccine provides valuable insights into the immune response elicited by inactivated virus vaccines. A study comparing anti-N levels found that naturally infected individuals had significantly higher anti-N IgG levels compared to those vaccinated with CoronaVac or BNT162b2 [45].

### 3.7. SARS-CoV-2 Anti-Spike and Anti-Nucleocapsid IgG Responses and Vaccine Type

**Figure 5:**
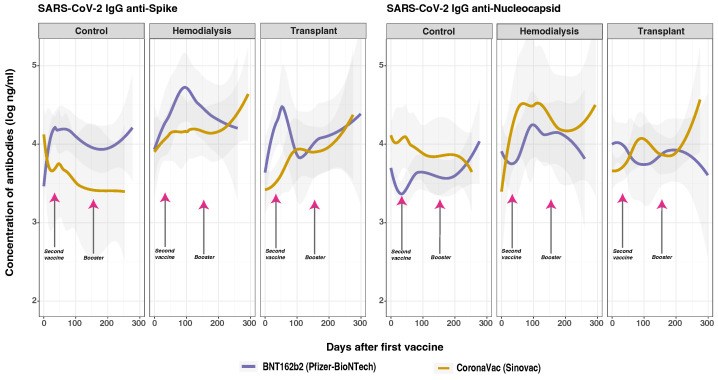
SARS-CoV-2 IgG anti-spike and anti-nucleocapsid protein distribution by cohort and vaccine type. Total anti-S and anti-N IgG concentrations were measured in control (*n* = 33), hemodialysis (*n* = 13), and transplant (*n* = 24) cohorts via multiplex assay. Day 0 refers to the day when the participants received their first vaccine dose. Arrows indicate mean times for second (36.6 ± 34.1 days) and booster vaccinations (154.3 ± 41.1 days). Among the subjects, 43 individuals (63.24%) were vaccinated with BNT162b2, while 25 individuals (36.76%) received CoronaVac. Additionally, 8 participants received homologous boosters, and 7 received heterologous boosters.

We next analyzed qualitative anti-S and anti-N IgG responses to BNT162b2 and CoronaVac, which revealed significant overlap in anti-N IgG levels within the TR group but not in the CO and HD groups (Figure 5). A key finding was that BNT162b2 resulted in higher anti-S IgG levels across the CO, HD, and TR groups, while CoronaVac was associated with higher anti-N IgG levels. However, no significant overall effect was observed based on the type of vaccine administered. Additionally, no significant interaction was found between prior infection and vaccine type for either anti-S or anti-N IgG levels.

A significant difference was found in anti-S IgG levels between cohorts vaccinated with CoronaVac and those with BNT162b2 (*p* < 0.050). Specifically, CoronaVac showed a significant impact on anti-S IgG responses between CO and HD groups (*p* < 0.050) and CO and TR groups (*p* < 0.010). Differences between the groups reveal that the CO group had the most stable anti-S IgG levels for both vaccines, with similar peak timings and gradual declines. BNT162b2 induced a stronger initial IgG response that declined over time in all the groups. CoronaVac showed a delayed but gradually increasing response in HD and TR, but not in CO. This variation suggests that underlying health conditions may influence the immune response in this population. Previous studies have shown that individuals vaccinated with the BNT162b2 vaccine had significantly higher neutralizing antibody titers compared to those who received the CoronaVac vaccine [46].

These effects were also exhibited in anti-N IgG levels when comparing the CO and HD groups (*p* < 0.050) and CO and TR groups (*p* < 0.001). Notably, CoronaVac elicited significantly higher anti-N IgG levels than BNT162b2 during the early stages of the immune response (0–15 days, *p* < 0.050). However, the effects on anti-N IgG levels between 15 and 35 days did not reach statistical significance. In the HD and TR patients who received the CoronaVac vaccine, while anti-N levels initially increased, anti-S levels peaked on day 300 (HD) and day 100 (TR), and anti-N levels decreased. This pattern of initially high anti-N levels gradually decreasing, followed by an increase in anti-S levels, could suggest recent infection rather than a response solely to vaccination. It could also be due to the composition of the CoronaVac vaccine, as attenuated viral components can potentially induce an anti-N IgG response that may boost natural infection, and vice versa, or vaccination [40,47].

As previously described in the GLMM (Table 3), many factors could affect anti-S and anti-N IgG levels among the vaccinated subjects, including prior infection, number of doses, and time of sampling after vaccination. Anti-N antibody measures might serve as a tool to identify past and current COVID-19 infections among a population vaccinated with mRNA-based vaccines. However, vaccination with CoronaVac may not be as effective, as attenuated virus vaccines could potentially elicit an anti-N response in addition to inducing antibodies against S protein. This could boost the immune response to natural infection or other types of vaccination [40].

## 4. Discussion

The COVID-19 pandemic profoundly impacted global health. Early vaccine trials excluded HD and TR [3,4]. Understanding the humoral response to SARS-CoV-2 infection and vaccination, along with vaccine and population-specific factors, is crucial for protecting vulnerable groups. While studies have reported immune responses in TR and HD after SARS-CoV-2 vaccination, data on mRNA and inactivated vaccines remain limited, especially outside the U.S. and Europe. Our study analyzed the antibody response to COVID-19 vaccination in 70 subjects across 3 cohorts: HD, TR, and CO, focusing on the BNT162b2 and CoronaVac vaccines (Figure 1). We applied mixed-effects modeling to assess variability in anti-S, anti-RBD, and anti-N IgG antibodies, focusing on response magnitude and duration. This approach provided more accurate estimates by accounting for both fixed effects (e.g., baseline subject characteristics) and random effects (e.g., cohort variability).

Our analysis of anti-S IgG, anti-RBD, and anti-N responses across different cohorts revealed significant differences, particularly within the first 130 days post-vaccination (Figure 3). While the CO group experienced an early peak in both anti-S and anti-RBD IgG, followed by stabilization, the TR group had a similar pattern, but with a slower rise and later peaks. The HD group, however, showed fluctuating levels, with an initial peak, a subsequent decline, and a later rise. Overall, the CO group maintained more stable antibody levels compared to the fluctuating levels observed in the HD and TR groups. The fluctuations in the HD and TR groups suggest that changes in anti-S and anti-RBD IgG levels are likely attributable to vaccination rather than reinfection. This finding supports the existing literature, which indicates that anti-RBD IgG levels generally remain stable with reinfection but increase with vaccination. This “back-boosting” effect occurs when additional vaccinations or exposure to different virus strains enhance the immune response by reactivating memory B and T cells.

Furthermore, the differences in anti-N IgG antibody responses between HD and TR compared to CO suggest that the CO group might have encountered new infections (Figure 3). The increase in anti-N IgG levels observed towards the end of the study, along with the decline in anti-S IgG levels from their peak on day 35, supports this possibility. The peak and subsequent decline in anti-S IgG levels observed in the CO cohort are consistent with established patterns of antibody kinetics in the general population [40]. The rise in anti-N levels on day 200, despite the decline in anti-S IgG, suggests a complex immunological response, possibly influenced by subsequent virus exposure. This aligns with findings in healthy individuals, where anti-N IgG peaks around 100 days post-infection and persists for months, with reinfection causing significant increases and vaccination having minimal impact over time [43].

The comparative analysis of anti-N levels also highlighted differences between the immunocompromised cohorts, with both the TR and HD groups showing higher concentrations than the CO group. However, the smaller increase in both anti-S and anti-N IgG levels in the TR group compared to the HD group may reflect the impact of immunosuppressive therapy on immune responses to infection and vaccination [41]. This observation aligns with existing literature suggesting that immunosuppressed individuals often exhibit a diminished vaccine-induced immune response [41]. These findings highlight the variability in immune responses across different patient groups and emphasize the need for vaccination strategies and monitoring approaches to address each group’s distinct immunological profile.

The GLMM analysis revealed that prior SARS-CoV-2 infection had a significant impact on anti-S IgG levels post-vaccination across all cohorts. Temporal variations in antibody peaks indicate that the individuals with prior infection tended to reach peak antibody levels earlier than those without a history of infection (Figure 4). The differences between the study groups revealed notable findings. In the HD group, prior infection significantly impacted both anti-S and anti-RBD early responses, resulting in higher anti-S IgG levels compared to the CO and TR groups. Although the CO and TR groups also showed elevated anti-S IgG levels in those with prior infection, their antibody concentrations displayed more variable peaks and fluctuations.

Our analysis indicates that individuals with a history of SARS-CoV-2 infection exhibited higher baseline anti-N levels and a significant increase over time. This outcome supports the understanding that individuals with prior infections have higher initial anti-N levels, as this is a key target of the immune response. Natural infection also appears to boost these levels more effectively than vaccination alone. Interestingly, while anti-N levels in the TR and CO groups decreased over time, the HD group maintained relatively stable levels. Specifically, the CO group, which showed higher anti-N levels compared to the HD and TR groups, likely represents those with more recent infections. Conversely, by day 100, both HD and TR groups, which were COVID-negative during the study, exhibited an increase in anti-N levels. This could suggest new infections or heightened immune responses in these groups. Additionally, individuals without prior infection in the HD and TR groups showed rising anti-N levels after day 100, whereas the CO group had stable anti-N levels, suggesting a more stable immune response and fewer new infections. This highlights the significant impact of prior infection on both the timing and magnitude of the anti-S IgG response across all cohorts.

Vaccine type also had a significant effect on the SARS-CoV-2 anti-S and anti-RBD IgG antibody responses, indicating that both the BNT162b2 and CoronaVac vaccines elicited robust humoral responses in all three cohorts. These responses were more pronounced after two doses and four weeks post-vaccination (Figure 5). BNT162b2 elicited higher anti-S and anti-RBD IgG levels at all time points in both the CO and TR groups compared to CoronaVac. This pattern did not hold for the HD group, where BNT162b2 maintained higher antibody levels up to day 100, after which they declined. Meanwhile, CoronaVac began to increase, eventually surpassing BNT162b2 by day 250. When comparing vaccine types and the anti-S and anti-RBD antibody responses between the cohorts, it was evident that the CO group showed the most stable and consistent antibody levels for both vaccines, with similar peak timings and gradual declines. The HD group exhibited a delayed peak for BNT162b2 and more fluctuations for CoronaVac, suggesting a variable immune response that was potentially influenced by the underlying health conditions [46]. The TR group demonstrated the greatest variability, with significant fluctuations in antibody levels for both vaccines, indicating an even more inconsistent immune response that was likely due to immunosuppressive treatments or other factors related to transplantation.

Vaccine type also had a significant effect on the anti-N IgG response, although BNT162b2 and CoronaVac both target the spike protein rather than the nucleocapsid protein (Figure 5). However, CoronaVac is an inactivated virus vaccine that may also stimulate the production of nucleocapsid antigens, likely explaining the higher levels of anti-N IgG antibodies seen in its recipients compared to those who received BNT162b2 [40,47]. This may reflect differences in vaccine-induced immunity compared to natural infection. Furthermore, significant interaction effects were observed in the HD and TR groups between prior SARS-CoV-2 infection and vaccine type. Both vaccines induced higher antibody levels in individuals with a previous infection at baseline in these groups. These findings highlight the complex interplay between patient-specific characteristics, prior infection status, and vaccine type in shaping the immune response. The combined effects of the two vaccines in heterologous boosters could result in complex immune responses that are distinct from those observed with homologous booster doses. However, our ability to assess the specific effects of each booster type is limited, primarily because the majority of the subjects received a heterologous combination of CoronaVac followed by BNT162b2 vaccines. As a result, isolating the effects of each individual booster has proven difficult due to a lack of sufficient data. Specifically, our study lacks an adequate sample size that received a homogeneous vaccination, which is essential for conducting a comparative analysis.

Additionally, our study has several other limitations, the most significant being the small sample size. Recruitment was constrained by the rapid vaccination rollout in the Dominican Republic, making it difficult to enroll unvaccinated subjects, especially among immunocompromised groups who were prioritized for vaccination. Vaccine hesitancy among hemodialysis patients, likely due to their deteriorating health, further reduced the sample size. Additionally, age differences between groups complicated our ability to control for confounding factors, making it hard to separate age effects from group effects. Lastly, a longer follow-up period is needed to assess long-term vaccine responses and the impact of different dosing schedules.

The study population was demographically diverse in age, balanced in sex, and primarily Hispanic or Latino, with a significant Black representation, reflecting the Dominican Republic’s COVID-19 context. The lower vaccination rate in the CO group compared to the two immunocompromised groups aligns with previous research indicating barriers or delays, which may be related to concerns about vaccine novelty, side effects, or a perceived lower risk of COVID-19 among healthy and younger individuals [48]. This finding mirrors global trends of vaccine hesitancy among individuals with chronic health conditions, particularly in HD, regarding the uptake of second and booster doses [49]. The reluctance in HD may stem from less-informed health beliefs, an underestimated risk of COVID-19, concerns about vaccine safety, or weakened patient–clinician relationships [49]. However, patients are more likely to become vaccinated if recommended by their nephrologist [49]. On the other hand, 91.7% of the TR patients received two or more doses. This high vaccination rate may be attributed to the higher education levels observed in this group, concerns about their clinical history, and a lower initial vaccine response [18]. Higher education often leads to better adherence to preventive measures and more proactive healthcare, which could positively influence their COVID-19 outcomes [50].

Overall, our results underscore the variability in immune responses to SARS-CoV-2 among different patient groups and highlight the critical role of vaccine type in modulating these responses, particularly the interactions of anti-S IgG between the HD, TR, and CO groups. This variability has important implications for real-world clinical applications, guiding vaccine selection for immunocompromised populations to recommend vaccines that elicit stronger immune defenses. Additionally, routine monitoring of anti-N IgG levels can help to assess new infections and vaccine efficacy, prompting timely booster doses for those at higher risk. Public health messaging can be enhanced by educating patients about the protective benefits of specific vaccines, while policymakers can prioritize distribution strategies based on these findings. Ultimately, continued research into these immunological responses will inform future vaccine designs, optimizing protection against SARS-CoV-2 variants and improving overall vaccination outcomes, especially for vulnerable populations.

## 5. Conclusions

The CO group had an early peak with stable anti-S and anti-RBD antibodies; the TR group showed a gradually lower increase; and the HD group exhibited fluctuating but generally high levels, likely due to back-boosting effects. However, the elevated anti-N IgG after a decline in anti-S suggests recent infection rather than vaccine response. Prior SARS-CoV-2 infection led to an early peak and higher anti-S IgG post-vaccination in all groups, but anti-N IgG levels were similar to those without prior infection. BNT162b2 was linked to higher anti-S IgG levels, while CoronaVac led to higher anti-N IgG levels. This international research collaboration identified vaccine-related factors influencing anti-S, anti-RBD, and anti-N IgG levels in HD and TR, offering insights to enhance public health strategies for protecting vulnerable populations.

## Figures and Tables

**Table 1:** The participant pool comprised a total of 70 individuals, distributed across three cohorts: control (*n* = 33), hemodialysis (*n* = 13), and transplant (*n* = 24). The groups were stratified based on the subjects’ prior history of COVID-19: positive (*n* = 31) or negative (*n* = 39). Statistical comparisons for categorical variables were performed using Pearson’s Chi-squared test and Fisher’s exact test. Categorical variables are presented as counts (percentages).

Characteristic	Control, *n* = 33 ^1^	Hemodialysis, *n* = 13 ^1^	Transplant, *n* = 24 ^1^	*p*-Value ^2^
**Age**				0.150
18–24	6 (18%)	1 (7.7%)	3 (13%)	
25–44	21 (64%)	5 (38%)	11 (46%)	
45–65	6 (18%)	7 (54%)	10 (42%)	
**Sex**				0.200
Female	19 (58%)	4 (31%)	10 (42%)	
Male	14 (42%)	9 (69%)	14 (58%)	
**Race**				<0.001
Asian	1 (3.0%)	0 (0%)	0 (0%)	
Black	15 (45%)	10 (77%)	24 (100%)	
White	7 (21%)	0 (0%)	0 (0%)	
Not reported	10 (30%)	3 (23%)	0 (0%)	
**Ethnicity**				0.009
Hispanic or Latino	19 (58%)	10 (77%)	19 (79%)	
Non-Hispanic or non-Latino	4 (12%)	0 (0%)	5 (21%)	
Not reported	10 (30%)	3 (23%)	0 (0%)	
**Education level**				<0.001
Less than high school	1 (3.0%)	5 (38%)	1 (4.2%)	
High school graduate	4 (12%)	3 (23%)	7 (29%)	
Higher education	9 (27%)	0 (0%)	15 (63%)	
Graduate education	3 (9.1%)	0 (0%)	0 (0%)	
Not reported	16 (48%)	5 (38%)	1 (4.2%)	
**Underlying conditions**				<0.001
None	27 (82%)	0 (0%)	1 (4.2%)	
At least one	6 (18%)	2 (15%)	6 (25%)	
Two or more	0 (0%)	11 (85%)	17 (71%)	
**COVID-19 positive before first fingerstick**				0.053
Yes	10 (30%)	6 (46%)	15 (63%)	
No	23 (70%)	7 (54%)	9 (38%)	

^1^ *n* (%); ^2^ Fisher’s exact test; Pearson’s Chi-squared test.

**Table 2:** A total of 271 capillary blood samples were collected from 70 subjects in three cohorts: control (*n* = 33), HD (*n* = 13), and TR (*n* = 24). Among them, 43 subjects were vaccinated with BNT162b2, including those who received homologous (*n* = 8) and heterologous boosters (*n* = 7), and 25 subjects were vaccinated with CoronaVac. Statistical comparisons for categorical variables were assessed using Pearson’s Chi-squared test and Fisher’s exact test.

Characteristic	Control, *n* = 33 ^1^	Hemodialysis, *n* = 13 ^1^	Transplant, *n* = 24 ^1^	*p*-Value ^2^
**Vaccine doses**				<0.001
One dose	11 (33%)	5 (38%)	2 (8.3%)	
Two doses	20 (61%)	4 (31%)	11 (46%)	
Fully vaccinated and one booster	2 (6.1%)	2 (15%)	10 (42%)	
Two or more boosters	0 (0%)	0 (0%)	1 (4.2%)	
Not reported	0 (0%)	2 (15%)	0 (0%)	
**Vaccine type (prime schedule)**				0.066
BNT162b2	25 (76%)	7 (64%)	11 (46%)	
CoronaVac	8 (24%)	4 (36%)	13 (54%)	
**Booster schedule**				0.700
Heterologous	0 (0%)	1 (50%)	6 (55%)	
Homologous	2 (100%)	1 (50%)	5 (45%)	
**COVID-19 positive before first fingerstick**				0.053
Yes	10 (30%)	6 (46%)	15 (63%)	
No	23 (70%)	7 (54%)	9 (38%)	
**COVID-19 positive during the study**				>0.900
Yes	8 (24%)	2 (15%)	5 (21%)	
Not reported	25 (76%)	11 (85%)	19 (79%)	

^1^ *n* (%); ^2^ Fisher’s exact test; Pearson’s Chi-squared test.

**Table 3:** A total of 11 independent variables were explored in association with the responses of the SARS-CoV-2 anti-Spike IgG antibody. Statistical significance was determined based on a 95% confidence interval (CI) for the standard coefficient (β).

Variable	β	95% CI ^1^	*p*-Value
**Time point**			
1	—	—	
2	0.42	−0.04, 0.89	0.076
3	0.63	0.17, 1.10	0.008
4	0.54	0.10, 0.98	0.018
5	0.90	0.43, 1.4	<0.001
**Cohort**			
Control	—	—	
Hemodialysis	1.30	−0.13, 2.80	0.072
Transplant	0.34	−0.53, 1.20	0.400
**Vaccine doses**	0.76	0.20, 1.30	0.008
**Age**			
18–24	—	—	
25–44	−0.52	−1.4, 0.35	0.200
45–65	0.41	−0.30, 1.1	0.300
**Sex**			
Female	—	—	
Male	−0.39	−1.10, 0.31	0.300
**Underlying conditions**	−0.98	−1.60, −0.40	0.001
**COVID-19 positive before first fingerstick**			
No	—	—	
Yes	1.10	0.23, 1.90	0.014
**Vaccine type (prime schedule)**			
BNT162b2	—	—	
CoronaVac	1.80	0.81, 2.80	<0.001
**Booster schedule**			
Heterologous	—	—	
Homologous	1.40	0.30, 2.50	0.014
**Education level**	−0.01	−0.68, 0.66	>0.900

^1^ CI = Confidence Interval.

## Data Availability

The original data presented in this study, along with the R code used for analysis, are openly available at FigShare.com (DOI 10.60593/ur.d.27460125).

## Supplementary Materials

The following supporting information can be downloaded at: https://www.mdpi.com/article/10.3390/vaccines12121312/s1, Figure S1. Correlation of SARS-CoV-2 IgG anti-spike protein and anti-RBD subunit by cohort and measured days after first vaccine; Figure S2. Correlation of SARS-CoV-2 IgG anti-spike protein and anti-S2 subunit by cohort and measured days after first vaccine; Figure S3. Correlation of SARS-CoV-2 IgG anti-RBD and anti-S2 subunits by cohort and measured days after first vaccine; Figure S4. Correlation of SARS-CoV-2 IgG anti-spike and anti-nucleocapsid proteins by cohort and measured days after first vaccine; Table S1. End-stage kidney disease and transplant cohort characteristics. This table presents the results of a statistical comparison of clinical characteristics between hemodialysis patients (*n* = 13) and transplant recipients (*n* = 24); Table S2. Hemodialysis characteristics. This table summarizes the hemodialysis characteristics of 13 subjects, focusing on access type and treatment duration; Table S3. Summary of transplant characteristics for 24 subjects.

## Abbreviations

The following abbreviations are used in this manuscript:

CO	Control group
CONABIOS	National Health Bioethics Committee of the Dominican Republic
	(the Spanish acronym)
COVID-19	Coronavirus disease 2019
DOAJ	Directory of Open Access Journals
ESRD	End-Stage Renal Disease
GLMM	Generalized Linear Mixed Models
HD	Hemodialysis Patients
Hgb	Hemoglobin
IgG	Immunoglobin G
INCORT	Instituto Nacional de Coordinación de Trasplante
IRB	Institutional Review Boards
MDPI	Multidisciplinary Digital Publishing Institute
mPlex-CoV	Fluorescent Multiplex Assay
N	Nucleocapsid
PCR	Polymerase Chain Reaction
RNA	Ribonucleic acid
S	Spike
SARS-CoV-2	Severe Acute Respiratory Syndrome Coronavirus 2
TR	Transplant Recipients
VAMS	Volumetric Micro Sampling

## References

[1] World Health Organization WHO Caronavirus Dashboard. https://covid19.who.int/.

[2] World Health Organization (2023). Status of COVID-19 Vaccines Within WHO EUL/PQ Evaluation Process. https://extranet.who.int/prequal/sites/default/files/document_files/Status_COVID_VAX_08AUgust2023.pdf.

[3] Carr E.J., Kronbichler A., Graham-Brown M., Abra G., Argyropoulos C., Harper L., Lerma E.V., Suri R.S., Topf J., Willicombe M. (2021). Systematic Review of Early Immune Response to SARS-CoV-2 Vaccination Among Patients with Chronic Kidney Disease. Kidney Int. Rep..

[4] Goffin E., Candellier A., Vart P., Noordzij M., Arnol M., Covic A., Lentini P., Malik S., Reichert L.J., Sever M.S. (2021). COVID-19 related mortality in kidney transplant and hemodialysis patients: A comparative, prospective registry based study. Nephrol. Dial. Transplant..

[5] Wijewickrama E.S., Abdul Hafidz M.I., Robinson B.M., Johnson D.W., Liew A., Dreyer G., Caskey F.J., Bello A.K., Zaidi D., Damster S. (2022). Availability and prioritisation of COVID-19 vaccines among patients with advanced chronic kidney disease and kidney failure during the height of the pandemic: A global survey by the International Society of Nephrology. BMJ Open.

[6] Cameron A., Porterfield C.A., Byron L., Wang C., Pearson Z., Bohrhunter J.L., Cardillo A.B., Ryan-Muntz L., Sorensen R.A., Caserta M. (2021). A Multiplex Microsphere IgG Assay for SARS-CoV-2 Using ACE2-Mediated Inhibition as a Surrogate for Neutralization. J. Clin. Microbiol..

[7] Sasikala M., Shashidhar J., Deepika G., Ravikanth V., Krishna V.V., Sadhana Y., Pragathi K., Reddy D.N. (2021). Immunological memory and neutralizing activity to a single dose of COVID-19 vaccine in previously infected individuals. Int. J. Infect. Dis..

[8] Wisnivesky J.P., Stone K., Bagiella E., Doernberg M., Mendu D.R., Lin J.J., Kale M. (2021). Long-term Persistence of Neutralizing Antibodies to SARS-CoV-2 Following Infection. J. Gen. Intern. Med..

[9] Anand S., Montez-Rath M.E., Han J., Garcia P., Cadden L., Hunsader P., Kerschmann R., Beyer P., Dittrich M., Block G.A. (2021). Antibody Response to COVID-19 Vaccination in Patients Receiving Dialysis. J. Am. Soc. Nephrol..

[10] Windpessl M., Bruchfeld A., Anders H.J., Kramer H., Waldman M., Renia L., Ng L.F.P., Xing Z., Kronbichler A. (2021). COVID-19 vaccines and kidney disease. Nat. Reviews. Nephrol..

[11] Bertrand D., Hamzaoui M., Lemée V., Lamulle J., Hanoy M., Laurent C., Lebourg L., Etienne I., Lemoine M., Le Roy F. (2021). Antibody and T Cell Response to SARS-CoV-2 Messenger RNA BNT162b2 Vaccine in Kidney Transplant Recipients and Hemodialysis Patients. J. Am. Soc. Nephrol..

[12] Marion O., Abravanel F., Couat C., Faguer S., Esposito L., Hebral A.L., Izopet J., Kamar N. (2021). Safety and Immunogenicity of Anti–SARS-CoV-2 Messenger RNA Vaccines in Recipients of Solid Organ Transplants. Ann. Intern. Med..

[13] Simon B., Rubey H., Treipl A., Gromann M., Hemedi B., Zehetmayer S., Kirsch B. (2021). Hemodialysis Patients Show a Highly Diminished Antibody Response after COVID-19 mRNA Vaccination Compared to Healthy Controls. Nephrol. Dial. Transplant..

[14] Ziemba R., Campbell K.N., Yang T.H., Schaeffer S.E., Mayo K.M., McGann P., Quinn S., Roach J., Huff E.D. (2021). Excess Death Estimates in Patients with End-Stage Renal Disease—United States, February-August 2020. MMWR Morb. Mortal. Wkly. Rep..

[15] Banham G.D., Godlee A., Faustini S.E., Cunningham A.F., Richter A., Harper L., Group o.b.o.t.C.H.B.S. (2021). Hemodialysis Patients Make Long-Lived Antibodies against SARS-CoV-2 that May Be Associated with Reduced Reinfection. J. Am. Soc. Nephrol..

[16] Boyarsky B.J., Werbel W.A., Avery R.K., Tobian A.A.R., Massie A.B., Segev D.L., Garonzik-Wang J.M. (2021). Antibody Response to 2-Dose SARS-CoV-2 mRNA Vaccine Series in Solid Organ Transplant Recipients. JAMA.

[17] Boyarsky B.J., Werbel W.A., Avery R.K., Tobian A.A.R., Massie A.B., Segev D.L., Garonzik-Wang J.M. (2021). Immunogenicity of a Single Dose of SARS-CoV-2 Messenger RNA Vaccine in Solid Organ Transplant Recipients. JAMA.

[18] Benotmane I., Gautier G., Perrin P., Olagne J., Cognard N., Fafi-Kremer S., Caillard S. (2021). Antibody Response After a Third Dose of the mRNA-1273 SARS-CoV-2 Vaccine in Kidney Transplant Recipients with Minimal Serologic Response to 2 Doses. JAMA.

[19] Ducloux D., Colladant M., Chabannes M., Yannaraki M., Courivaud C. (2021). Humoral response after three doses of BNT162b2 mRNA COVID-19 vaccine in patients on hemodialysis. Kidney Int..

[20] Kamar N., Abravanel F., Marion O., Couat C., Izopet J., Del Bello A. (2021). Three Doses of an mRNA Covid-19 Vaccine in Solid-Organ Transplant Recipients. N. Engl. J. Med..

[21] Han A., Min S., Jo E.A., Lee H., Kim Y.C., Han S.S., Kang H.G., Ahn Y.H., Oh I., Song E.Y. (2023). Association between Low Anti-spike Antibody Levels After the Third Dose of SARS-CoV-2 Vaccination and Hospitalization due to Symptomatic Breakthrough Infection in Kidney Transplant Recipients. Ann. Lab. Med..

[22] Weiss A., Hendrickx R., Stensgaard E., Jellingsø M., Sommer M.O. (2023). Kidney Transplant and Dialysis Patients Remain at Increased Risk for Succumbing to COVID-19. Transplantation.

[23] Barreiro P., Candel F.J., Carretero M.M., San Román J. (2023). Risk of severe COVID in solid organ transplant recipient. Rev. Esp. Quimioter..

[24] Chiang T.P.Y., Abedon A.T., Alejo J.L., Segev D.L., Massie A.B., Werbel W.A. (2023). Incident COVID-19 and Hospitalizations by Variant Era Among Vaccinated Solid Organ Transplant Recipients. JAMA Netw. Open.

[25] Pinchera B., Buonomo A.R., Trucillo E., Susini S., D’Agostino A., Di Filippo I., Tanzillo A., Villari R., Carrano R., Troisi R.I. (2023). COVID-19 in solid organ transplant recipients after 2 years of pandemic: Outcome and impact of antiviral treatments in a single-center study. Front. Transplant..

[26] Fernandez C. (2023). En República Dominicana, 5275 Pacientes Reciben Hemodiálisis y 500 Esperan un Riñón. Diario Libre.

[27] Instituto Nacional de Coordinación de Trasplante (INCORT) (2024). Trasplantes de órganos y Tejidos de la República Dominicana 2008–2023. Incort. Santo Domingo, Dominican Republic.

[28] Ministerio de Salud Pública de La República Dominicana (2021). Gobierno Presenta Plan Nacional de Vacunación Contra COVID-19. https://dominicantoday.com/dr/health/2021/02/13/vaccines-that-the-country-will-use-are-very-effective/.

[29] Dominican Today (2021). Vaccines That the Dominican Republic Will Use Are Very Effective. https://dominicantoday.com/dr/health/2021/02/13/vaccines-that-the-country-will-use-are-very-effective/.

[30] Pecora N.D., Zand M.S. (2020). Measuring the Serologic Response to Severe Acute Respiratory Syndrome Coronavirus 2: Methods and Meaning. Clin. Lab. Med..

[31] Wang J., Li D., Zhou Q., Wiltse A., Zand M.S. (2021). Antibody Mediated Immunity to SARS-CoV-2 and Human Coronaviruses: Multiplex Beads Assay and Volumetric Absorptive Microsampling to Generate Immune Repertoire Cartography. Front. Immunol..

[32] Kompaniyets L., Pennington A.F., Goodman A.B., Rosenblum H.G., Belay B., Ko J.Y., Chevinsky J.R., Schieber L.Z., Summers A.D., Lavery A.M. (2021). Underlying Medical Conditions and Severe Illness Among 540,667 Adults Hospitalized with COVID-19, March 2020–March 2021. Prev. Chronic. Dis..

[33] Wang J., Li D., Wiltse A., Emo J., Hilchey S.P., Zand M.S. (2019). Application of volumetric absorptive microsampling (VAMS) to measure multidimensional anti-influenza IgG antibodies by the mPlex-Flu assay. J. Clin. Transl. Sci..

[34] Bursac Z., Gauss C.H., Williams D.K., Hosmer D.W. (2008). Purposeful selection of variables in logistic regression. Source Code Biol. Med..

[35] United Nations Population Fund (UNFPA) (2022). Encuesta Nacional de Autopercepción Racial y Étnica en República Dominicana. https://dominicanrepublic.unfpa.org/es/publications/breve-encuesta-nacional-de-autopercepcion-racial-y-etnica-en-republica-dominicana.

[36] Narongkiatikhun P., Noppakun K., Chaiwarith R., Winichakoon P., Vongsanim S., Suteeka Y., Pongsuwan K., Kusirisin P., Wongsarikan N., Fanhchaksai K. (2023). Immunogenicity and Safety of Homologous and Heterologous Prime-Boost of CoronaVac^®^ and ChAdOx1 nCoV-19 among Hemodialysis Patients: An Observational Prospective Cohort Study. Vaccines.

[37] Pérez-Then E., Lucas C., Monteiro V.S., Miric M., Brache V., Cochon L., Vogels C.B.F., Malik A.A., De la Cruz E., Jorge A. (2022). Neutralizing antibodies against the SARS-CoV-2 Delta and Omicron variants following heterologous CoronaVac plus BNT162b2 booster vaccination. Nat. Med..

[38] Ramos A., Cardoso M.J., Ribeiro L., Guimarães J.T. (2022). Assessing SARS-CoV-2 Neutralizing Antibodies after BNT162b2 Vaccination and Their Correlation with SARS-CoV-2 IgG Anti-S1, Anti-RBD and Anti-S2 Serological Titers. Diagnostics.

[39] Anichini G., Terrosi C., Gandolfo C., Gori Savellini G., Fabrizi S., Miceli G.B., Cusi M.G. (2021). SARS-CoV-2 Antibody Response in Persons with Past Natural Infection. N. Engl. J. Med..

[40] Xu K., Dai L., Gao G.F. (2021). Humoral and cellular immunity and the safety of COVID-19 vaccines: A summary of data published by 21 May 2021. Int. Immunol..

[41] Stumpf J., Siepmann T., Lindner T., Karger C., Schwöbel J., Anders L., Faulhaber-Walter R., Schewe J., Martin H., Schirutschke H. (2021). Humoral and cellular immunity to SARS-CoV-2 vaccination in renal transplant versus dialysis patients: A prospective, multicenter observational study using mRNA-1273 or BNT162b2 mRNA vaccine. Lancet Reg. Health Eur..

[42] Rouphael N., Bausch-Jurken M. (2023). COVID-19 Vaccination Among Patients Receiving Maintenance Renal Replacement Therapy: Immune Response, Real-World Effectiveness, and Implications for the Future. J. Infect. Dis..

[43] Jarlhelt I., Pérez-Alós L., Bayarri-Olmos R., Hansen C.B., Petersen M.S., Weihe P., Armenteros J.J.A., Madsen J.R., Nielsen J.P.S., Hilsted L.M. (2023). Distinguishing SARS-CoV-2 infection and vaccine responses up to 18 months post-infection using nucleocapsid protein and receptor-binding domain antibodies. Microbiol. Spectr..

[44] Aguilar-Bretones M., Fouchier R.A.M., Koopmans M.P.G., van Nierop G.P. (2023). Impact of antigenic evolution and original antigenic sin on SARS-CoV-2 immunity. J. Clin. Investig..

[45] Qaqish A., Abbas M.M., Al-Tamimi M., Abbas M.A., Al-Omari M., Alqassieh R. (2022). SARS-CoV-2 Antinucleocapsid Antibody Response of mRNA and Inactivated Virus Vaccines Compared to Unvaccinated Individuals. Vaccines.

[46] Eren Sadioğlu R., Demir E., Evren E., Aktar M., Şafak S., Artan A.S., Meşe S., Ağaçfidan A., Çınar G., Önel M. (2021). Antibody response to two doses of inactivated SARS-CoV-2 vaccine (CoronaVac) in kidney transplant recipients. Transpl. Infect. Dis..

[47] Ibrahim K.Y., Moreira R.M., Santos C.F.D., Strabelli T.M.V., Belizário J.C., Pinto M.I.M., Marinho A., Pereira J.M., Mello L.S., Ando M.C. (2024). Immunogenicity of COVID-19 adsorbed inactivated vaccine (CoronaVac) and additional doses of mRNA BNT162b2 vaccine in immunocompromised adults compared with immunocompetent persons. Rev. Inst. Med. Trop. São Paulo.

[48] Lin C., Tu P., Beitsch L.M. (2021). Confidence and Receptivity for COVID-19 Vaccines: A Rapid Systematic Review. Vaccines.

[49] Wallace H., Mount P.F. (2022). COVID-19 beliefs and vaccination uptake in dialysis patients: Lessons from an anonymous patient survey. Intern. Med. J..

[50] Fisher K.A., Bloomstone S.J., Walder J., Crawford S., Fouayzi H., Mazor K.M. (2020). Attitudes Toward a Potential SARS-CoV-2 Vaccine: A Survey of U.S. Adults. Ann. Intern. Med..

