# Supplementary material for: Antibody Response to SARS-CoV-2 Vaccines in Transplant Recipients and Hemodialysis Patients: Data from the Dominican Republic
Source: Vaccines (Basel). 2024 Nov 23;12(12):1312. doi: 10.3390/vaccines12121312 (PMC11680170; doi:10.3390/vaccines12121312)
Supplement: Supplementary file 1 [file vaccines-12-01312-s001.zip › vaccines-3257450-supplementary.pdf]

Supplementary Figures and Tables:

Antibody Response to SARS-CoV-2 Vaccines in Transplant Recipients and Hemodialysis Patients: Data from the Dominican Republic

Lisette Alcantara S.[1], Eloy Alvarez Guerra[2], Dongmei Li[1], Samantha King[3], Shannon P. Hilchey[3], Qian Zhou[3], Stephen Dewhurst[4], Kevin Fiscella[5], and Martin S. Zand[1,3,6]

[1] Clinical and Translational Science Institute, University of Rochester, Rochester NY
[2] Instituto Nacional de Coordinación de Trasplante, and Instituto de Medicina Tropical y Salud Global, Universidad Iberoamericana, Santo Domingo, Dominican Republic
[3] Department of Medicine, Division of Nephrology, University of Rochester, Rochester NY
[4] Department Microbiology and Immunology, University of Rochester, Rochester NY
[5] Department of Family Medicine, University of Rochester, Rochester NY
[9] Department of Public Health Sciences, University of Rochester, Rochester NY

S1 Clinical Variables

Characteristic	Hemodialysis, N = 13[1]	Transplant, N = 24[1]	p-value[2]
**Age**			0.312
18-24	1 (7.7%)	3 (12.5%)	
25-44	5 (38.5%)	11 (45.8%)	
45-65	7 (53.8%)	10 (41.7%)	
**Sex**			0.225
Female	4 (30.8%)	10 (41.7%)	
Male	9 (69.2%)	14 (58.3%)	
**Vaccine type (Prime schedule)**			0.03
BNT162b2	7 (63.6%)	11 (45.8%)	
CoronaVac	4 (36.4%)	13 (54.2%)	
Not Reported	-	-	
**Booster schedule**			0.767
Heterologous	1 (50.0%)	6 (54.5%)	
Homologous	1 (50.0%)	5 (45.5%)	
Not Reported	-	-	
**Covid positive before first fingerstick**			0.314
Yes	6 (46.2%)	15 (62.5%)	
No	7 (53.8%)	9 (37.5%)	
**Covid positive during the study**			0.523
Yes	2 (15.4%)	5 (20.8%)	
Not Reported	11 (84.6%)	19 (79.2%)	
**Hospitalization**			0.031
Yes	3 (23.1%)	1 (4.2%)	
No	-	-	
**Ventilator use**			0.459
Yes	1 (7.7%)	0 (0%)	
No	-	-	
**Cause of ESRD**			<0.001
Hypertension (HTN) w/o other	5 (38.5%)	8 (33.3%)	
HTN and Pregnancy	2 (15.4%)	2 (8.3%)	
Diabetes Mellitus (DM)	2 (15.4%)	2 (8.3%)	
Congenital Anomalies	-	3 (12.5%)	
Rheumatic Heart Disease	-	2 (8.3%)	
Glomerulopathy	1 (7.7%)	5 (20.8%)	
Lupus	-	1 (4.2%)	
Others	3 (23.1%)	1 (4.2%)	
**Flu vaccine after dialysis or transplant**			<0.001
Yes	4 (30.8%)	21 (87.5%)	
No	-	-	
**Hepatitis B vaccine**			<0.001
Yes	7 (53.8%)	24 (100.0%)	
No	-	-	

[1] n (%) [2] Fisher's exact test; Pearson's Chi-squared test

Table S1: **Cohort characteristics**. Statistical comparison of clinical characteristics between hemodialysis patients (N=13) and transplant recipients (N=24), using Chi-squared and Fisher's exact tests. Age distribution and sex did not show significant differences between the groups (p = 0.312 and p = 0.225, respectively). However, significant differences were found in vaccine type (p =0 .03), hospitalization (p = 0.031), cause of ESRD (p < 0.001), flu vaccine uptake post-dialysis or transplant (p < 0.001), and Hepatitis B vaccination (p < 0.001). HD had a higher proportion of BNT162b2 vaccinations, while TR more commonly received the CoronaVac vaccine. Hospitalization rates were higher among the HD group. The cause of ESRD also differed significantly, with more cases of congenital anomalies and glomerulopathy in the TR group. Flu and Hepatitis B vaccine rates were significantly higher in TR. Other variables such as COVID-19 infection before and during the study, ventilator use, and booster schedules did not show significant differences.

Characteristic	Hemodialysis, N = 13
**Hemodialysis access**	
AV Fistula	4 (30.8%)
Catheter	3 (23.1%)
Catheter & AV Fistula	4 (30.8%)
Not reported	2 (15.4%)
**Dialysis vintage, mo**	
<12 months	1 (7.7%)
13- 24 months	7 (53.8%)
25 - 48 months	3 (23.1%)
>49 months	1 (7.7%)
Not reported	1 (7.7%)

Table S2: **Hemodialysis cohort characteristics**. This table summarizes the hemodialysis characteristics of 13 subjects, focusing on access type and treatment duration. Among them, 4 subjects (30.8%) used an AV fistula, 3 subjects (23.1%) used a catheter, and 4 subjects (30.8%) used both types of access. Most subjects (53.8%) had been on dialysis for 1-2 years, with the remaining subjects having either shorter or longer duration. Overall, the AV fistula was the most commonly used access method.

Characteristic	Transplant, N = 24
**Transplant time**	
<6 months	2 (8.30%)
7-12 months	3 (12.50%)
13 - 24 months	3 (12.50%)
>25 months	16 (66.70%)
**Induction therapy <12 Months**	
Basiliximab	4 (16.70%)
Thymoglobulin	2 (8.30%)
**Immunosupression (current)**	
Certican (Everolimus)	17 (70.80%)
Cyclosporine	19 (79.20%)
Mycophenolate Mofetil	9 (37.50%)
Prednisone	6 (25.00%)
Tacrolimus	9 (37.50%)
**Type of organ donation**	
Living donation	16 (66.70%)
Deceased donation	8 (33.30%)
**Organ rejection (Transplant 2020 - 2021)**	
Acute graft	2 (8.30%)
Chronic graft	1 (4.20%)
**Rejection treatment**	
Rituximab	2 (8.30%)
Tacrolimus	3 (12.50%)
Not reported	2 (8.30%)

Table S3: **Transplant corhort Characteristics.** The table summarizes transplant characteristics for 24 subjects. Most had their transplant over 3 years ago (66%). The most common immunosuppressants were Cyclosporine (79.2%) and Certican (70.8%). Living donations made up 66.7% of transplants. In 2020-2021, 8.3% experienced acute rejection and 4.2% had chronic rejection, with Tacrolimus (12.5%) and Rituximab (8.3%) used for rejection treatment.

S2 Correlations

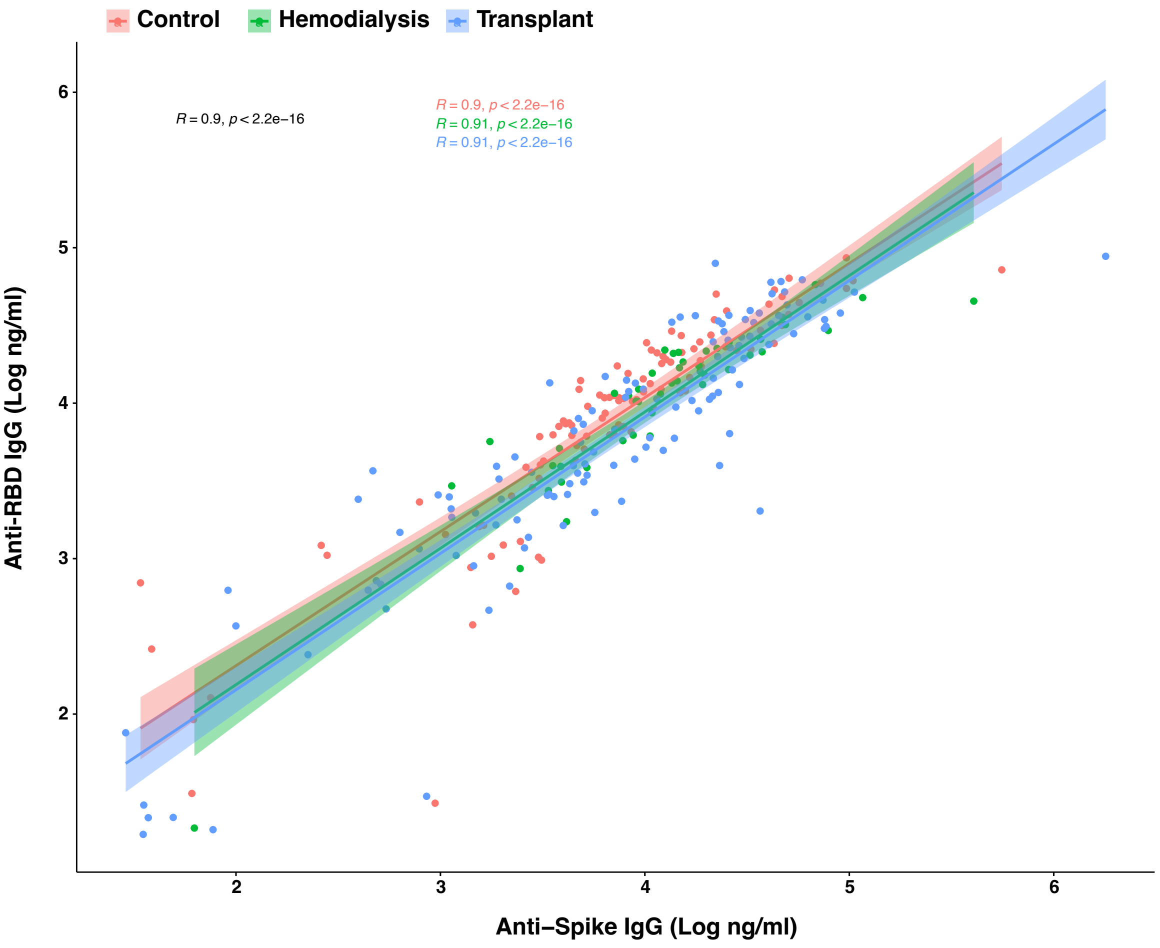

Figure S1: **Correlation of SARS-CoV-2 IgG anti-Spike protein and anti-RBD subunit by cohort and measured days after first vaccine.** The scatter plot shows a very strong positive correlation between anti-S and anti-RBD, with a Pearson coefficient of R = 0.90 (95% CI: 0.879 to 0.923, $p < 0.001$). All three groups exhibit strong positive correlations: CO (R = 0.90), HD (R = 0.91), and TR (R = 0.91), all statistically significant.

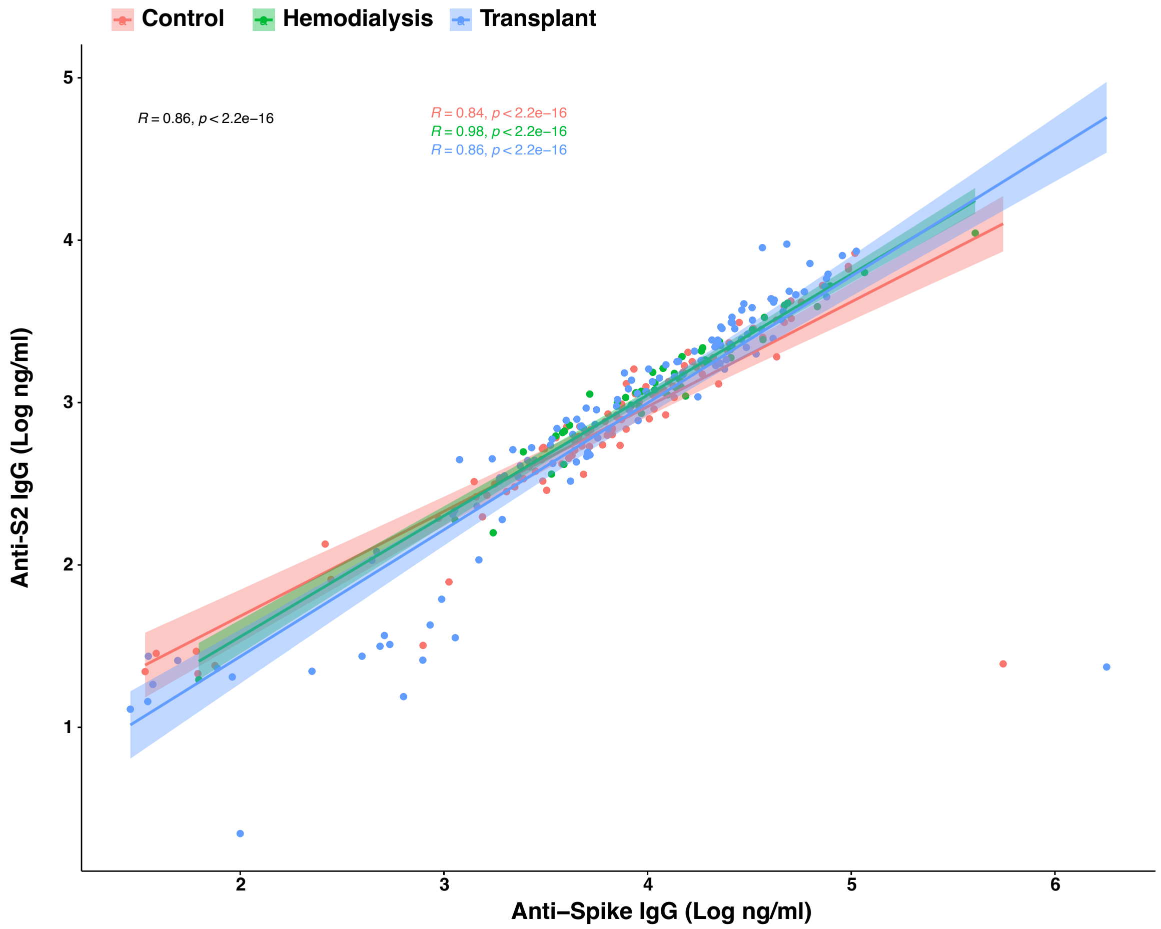

Figure S2: **Correlation of SARS-CoV-2 IgG anti-Spike protein and anti-S2 subunit by cohort and measured days after first vaccine.** The scatter plot shows a strong positive correlation between anti-S and the anti-S2 subunit, with a Pearson correlation coefficient of R = 0.86 (95% CI: 0.831 to 0.892, p < 0.001). All three groups demonstrate statistically significant positive correlations: the CO group (R = 0.84), the HD group (R = 0.98), and the TR group (R = 0.986).

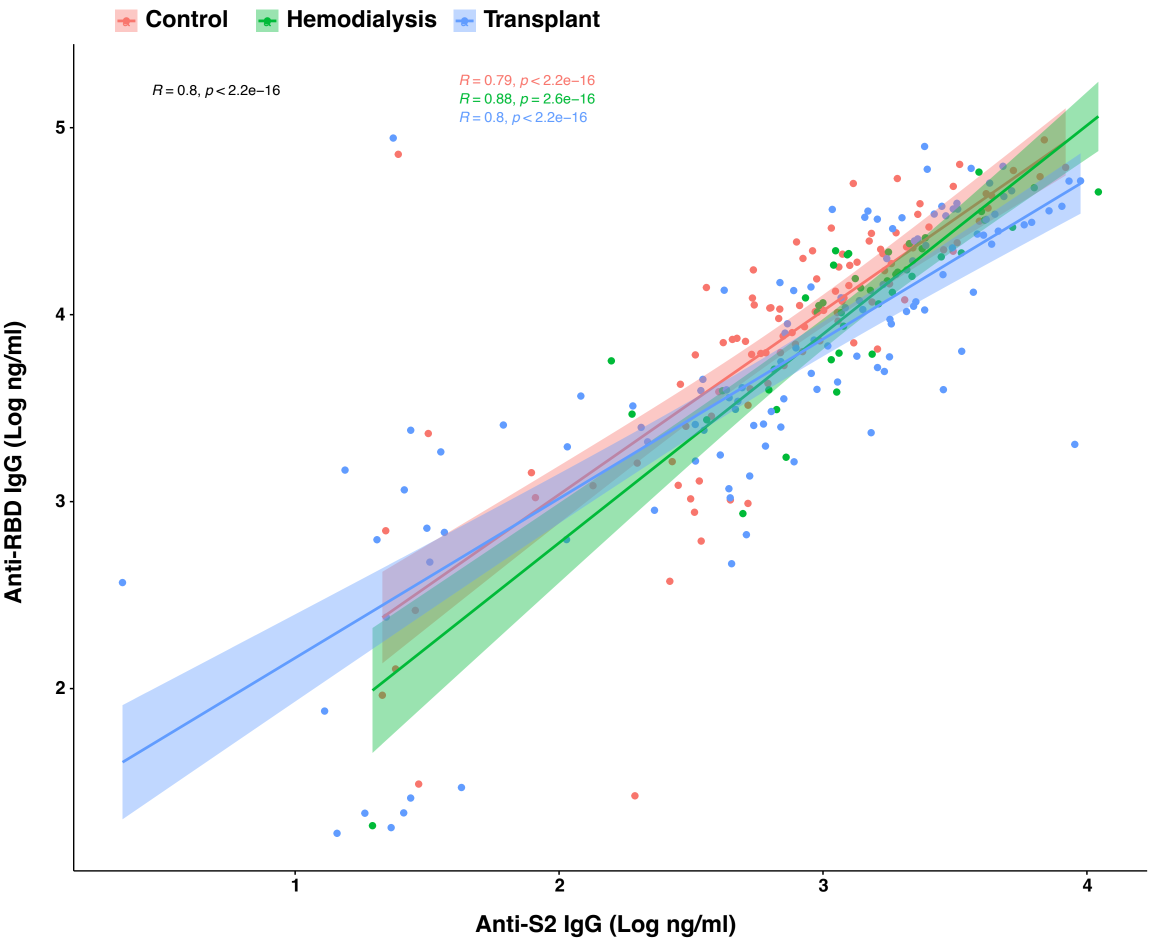

Figure S3: **Correlation of SARS-CoV-2 IgG anti-RBD and anti-S2 subunits by cohort and measured days after first vaccine.** The scatter plot shows a strong positive correlation between the anti-S2 and anti-RBD subunits, with a Pearson correlation coefficient of R = 0.80 (95% CI: 0.754 to 0.841, p < 0.001). All three groups exhibit statistically significant positive correlations: the CO group (R = 0.79), the HD group (R = 0.88), and the TR group (R = 0.80).

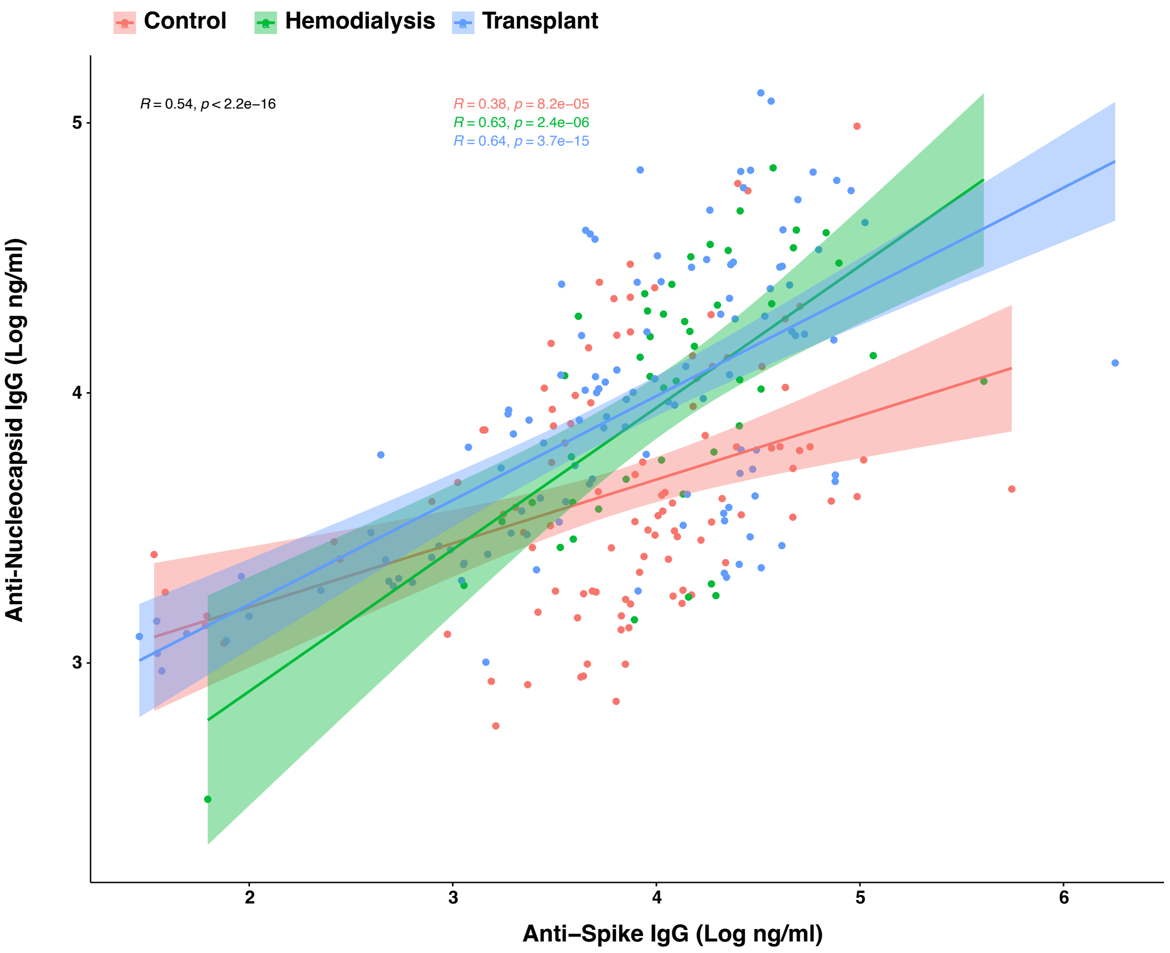

Figure S4: **Correlation of SARS-CoV-2 IgG anti-Spike and anti-Nucleocapsid proteins by cohort and measured days after first vaccine.** The scatter plot shows the correlation between anti-S and anti-N levels. The Pearson correlation coefficient is R = 0.54 (95% CI: 0.445 to 0.615, $p < 0.001$), indicating a moderate positive relationship. Among the groups, the CO group has a weaker correlation (R = 0.38), the HD group shows a stronger correlation (R = 0.63), and the TR group exhibits the highest correlation (R = 0.64). This analysis reveals varying strengths of association, with TR showing the strongest and CO the weakest correlation, although all are statistically significant.

References

Cook, O. E., G. H. Collaborator, and A. Q. Expert. 2015. Data from: Template and guidelines for using LaTeX in *The American Naturalist*. American Naturalist, Dryad Digital Repository, https://dx.doi.org/10.5061/dryad.XYZAB123.

Darwin, C. 1859. On the origin of species by means of natural selection, or the preservation of favoured races in the struggle for life. J. Murray, London.

Davis, E. B., K. A. Brakora, and A. H. Lee. 2011. Evolution of ruminant headgear: a review. Proceedings of the Royal Society B 278:2857–2865.

Inglis, R. F., P. G. Roberts, A. Gardner, and A. Buckling. 2011. Spite and the scale of competition in *Pseudomonas aeruginosa*. American Naturalist 178:276–285.

Fastovsky, D. E. 2009. Ideas in dinosaur paleontology: resonating to social and political context. Pages 239–253 *in* D. Sepkoski and M. Ruse, eds. The Paleobiological Revolution. University of Chicago Press, Chicago IL.

Xiao, X., D. J. McGlinn, and E. P. White. 2015. A strong test of the maximum entropy theory of ecology. American Naturalist 185:E705–E80.
